# Synthesis and Biological Evaluation of Novel Bufalin Derivatives

**DOI:** 10.3390/ijms23074007

**Published:** 2022-04-04

**Authors:** VishnuPriya Sampath, Noa Horesh, Ben Sasi, Hiba Zannadeh, Ilana Pogodin, Shiv Vardan Singh, Joseph Deutsch, David Lichtstein

**Affiliations:** 1 Department of Medical Neurobiology, Institute for Medical Research Israel-Canada, Faculty of Medicine, The Hebrew University-Hadassah Medical School, Jerusalem 91120, Israel; vishnulifebt@yahoo.com (V.S.); noa.rosenthal1@mail.huji.ac.il (N.H.); ben.sasi@mail.huji.ac.il (B.S.); hiba.hani1999@gmail.com (H.Z.); ilana.pogodin@mail.huji.ac.il (I.P.); 2 Department of Biochemistry, Faculty of Science, University of Allahabad, Prayagraj 211002, India; vardanshiva@gmail.com; 3 Department of Medicinal Chemistry, Institute of Drug Research, School of Pharmacy, Faculty of Medicine, The Hebrew University-Hadassah Medical School, Jerusalem 91120, Israel

**Keywords:** bufalin, cardiac steroids, ouabain, digoxin, cytotoxicity, heart failure, positive inotropy, Na^+^, K^+^-ATPase, cancer

## Abstract

Bufalin and other cardiac steroids (CS) have been used for centuries for the treatment of congestive heart failure, arrhythmias, and other maladies. However, toxicity and the small therapeutic window of this family of steroids limit their use. Therefore, attempts to synthesize a potent, but less toxic, CS are of major importance. In the present study, two novel bufalin derivatives were synthesized and some of their pharmacological properties were characterized. The reaction of bufalin with Ishikawa’s reagent resulted in the production of two novel bufalin derivatives: bufalin 2,3-ene and bufalin 3,4-ene. The compounds were purified with TLC and HPLC and their structure was verified with UV, NMR, and MS analyses. The biological activities of these compounds were evaluated by testing their ability to inhibit the Na^+^, K^+^-ATPase activity of the brain microsomal fraction to induce cytotoxic activity against the NCI-60 human tumor cell line panel and non-cancer human cells, and to increase the force of contraction of quail embryonic heart muscle cells in culture. The two steroids exhibited biological activities similar to those of other CS in the tested experimental systems, but with reduced cytotoxicity, advocating their development as drugs for the treatment of heart failure and arrhythmias.

## 1. Introduction

Bufalin, the major component of the traditional Chinese medicine Chan-Su, is an extract of the skin and parotid venom glands of a toad of the Bufo family [1]. Chan-Su has been widely used in China and other Asian countries to treat cancer and additional ailments [2]. Bufalin belongs to the cardiac steroid (CS) family and, like other members such as ouabain and digoxin, increases the force of contraction of heart muscle, thus improving circulation in cases of insufficient cardiac output [3]. However, the toxicity and the small therapeutic window of this family of steroids limits their use as cardiotonic drugs [4]. A similar problem is encountered in the use of these steroids for the treatment of cancer. Although bufalin has been shown to kill various tumor cells in vitro, it produced unsatisfactory results when administered in vivo, [5,6]. Due to its fast metabolism, toxicity, insolubility in water, and short half-life, its application in the clinical setting is limited [7]. In addition, bufalin and other CS have been shown to have potent anti-inflammatory and anti-viral activities [8,9,10]. However, all these beneficial properties are obtained at concentrations higher than the toxic effects of these compounds. Therefore, the development of bufalin derivatives with lower toxicity is of great importance.

The plasma membrane Na^+^ and K^+^ transporter Na^+^, K^+^-ATPase [11] is an established receptor for CS. The interaction of these steroids with Na^+^, K^+^-ATPase results in inhibition of the ion-pumping function and, in addition, causes the activation of several signal transduction cascades, including mitogen-activated protein kinase; extracellular signal-regulated kinase; proto-oncogene tyrosine-protein kinase (Src); and PI3K/Akt, Ca^++^ signaling, and reactive oxygen species generation pathways [12,13]. It is well established that the toxicity of CS in the heart is due to calcium overload, produced by excessive inhibition of the Na^+^, K^+^-ATPase in the myocytes, leading to arrhythmia and lethality [14]. Conversely, the positive inotropic effect, as well as the anti-cancer and anti-viral effects, are largely a result of the CS-induced signaling activation. Indeed, the inhibition of ERK activation totally prevented the bufalin and other CS-induced increase in heart contractility [15]; the bufalin anti-cancer effect was shown repeatedly to be mediated by ERK and AKT signaling [16], as was the anti-viral activity of CS [17]. It is reasonable to suggest, therefore, that differences in CS-induced signaling by various CS will have a profound effect on their pharmacological profiles.

CS are composed of three major structural components: a steroid core, in which rings AB and CD are cis-fused, whereas rings BC are trans-fused; a 5- and 6-membered lactone ring at position 17 (cardenolides and bufadienolides, respectively); and a variable number of sugar residues at position 3. The significance of the moiety and orientation at position 3 of the steroid for its biological activity was studied extensively, especially in relation to the nature of the sugar bound at this position [18]. Furthermore, our previous study showed that the/orientation of the 3-OH group CS may have a substantial effect on biological activity. Whereas the 3-OH isomer displayed the standard capability of increasing heart contractility, a 3-OH isomer did not boost the force of contraction, but actually inhibited the contractility induced by digoxin [19].

In the present study, two isomers lacking an OH at the 3 position, bufalin-2,3-ene and bufalin-3,4-ene, were synthesized. The biological activities of these compounds were evaluated by testing their ability to inhibit Na^+^, K^+^-ATPase activity in a pig brain microsomal fraction, to cause cytotoxicity in human cancer cells and to prompt positive inotropy in quail cardiac cells in culture. 

## 2. Results

### 2.1. Synthesis and Structure Validation of Bufalin Derivatives

**Figure 1:**
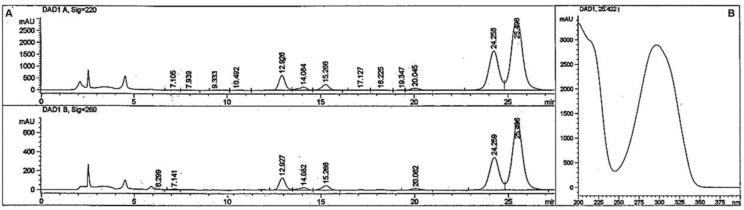
Elution profile of reaction mixture on high performance liquid chromatography, C18 column. Aliquots of the partially purified material obtained from the TLC column (see Materials and Methods) were dissolved in methyl alcohol (0.3 mL) and a 50 µL sample was injected onto a Luna C-18, 5 μm column (250 × 4.6 mm) provided with a pre-column. Elution was performed with a 35 min, 68% CH3CN/water isocratic system with a flow rate of 1.0 mL/min. The lines represent the absorbance at 220 nm (**A**, upper panel) and at 260 nm (**A**, lower panel). The absorbance of the peak at 25.4 min retention time at different wavelengths is shown in (**B**).

The bufalin derivatives were synthesized with Ishikawa’s reagent, which is typically used to convert alcohols into alkyl fluorides and carboxylic acids into acyl fluorides [20], as described in chemical experimental procedures (Section 4.5). A suspension of bufalin (15 mg, 0.039 mmol) in dry diethyl ether (1.0 mL) was prepared in a 5 mL glass vial with a magnetic stirrer. The vial was cooled at 8 °C, wrapped in aluminum foil, and Ishikawa’s reagent (70 mg, 0.310 mmol, 60 μL) was added slowly. The mixture was stirred for 2 h at 8 °C and overnight at room temperature. To separate the product from excess reagent, the solution was loaded on a silica gel thin-layer chromatography (TLC) plate and eluted with 70% diethyl ether and hexane for 4 min. Following visualization with a UV lamp at 260 nm, the silica gel plate at 2 cm was scraped into a small vial and the organic material was dissolved in methanol (0.3 mL). A clear suspension from this solution (50 μL) was injected into the high-performance liquid chromatography (HPLC) system, producing the pattern shown in Figure 1. Peaks with an absorbance at λ max 300 nm at 24–25 and 25–26 min retention time were collected, dried (0.8 mg, 5.3% yield), and subjected to mass spectrometry (MS), nuclear magnetic resonance (NMR), and X-Ray crystallographic measurements.

**Figure 2:**
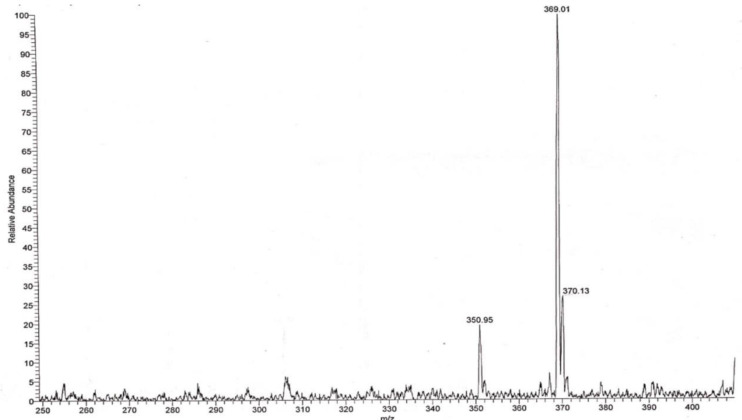
Mass spectrum analysis of compound 2. After collecting compound 2 (retention time 25–26 min in HPLC), sample was evaporated, the product was dissolved in 0.3 mL methyl alcohol, and 5 µL was subjected to mass spectrum analysis. The electron spray mass spectrum was obtained with a TSQ Quantum Access mass spectrometer (Thermo Fisher Scientific, Basel, Switzerland).

Surprisingly, as can be seen in Figure 1, purification of the products with preparative HPLC produced two different solid compounds (ratio of about 1:2) with no traces of fluorine atoms (measured with Fluorine NMR spectroscopy). The first product (retention time 24–25 min, compound 1) appeared as an amorphous solid powder (m.p. 165–168 °C). The second product (retention time 25–26 min, compound 2) formed large crystals with an almost identical melting point (m.p. 166–168 °C). The mixture of the two compounds, however, exhibited a lower melting point of more than 30°, indicating that the two compounds are not identical. The two compounds had an identical mass spectrum, with a protonated molecular mass of 369 without mass corresponding to fluorine atom (Figure 2). This molecular mass may correspond to isomers of dehydroxy bufalin formed by the replacement of one of the OH groups at the C3 or C14 position with a hydrogen atom from a nearby position (bufalin–H_2_O).

**Figure 3:**
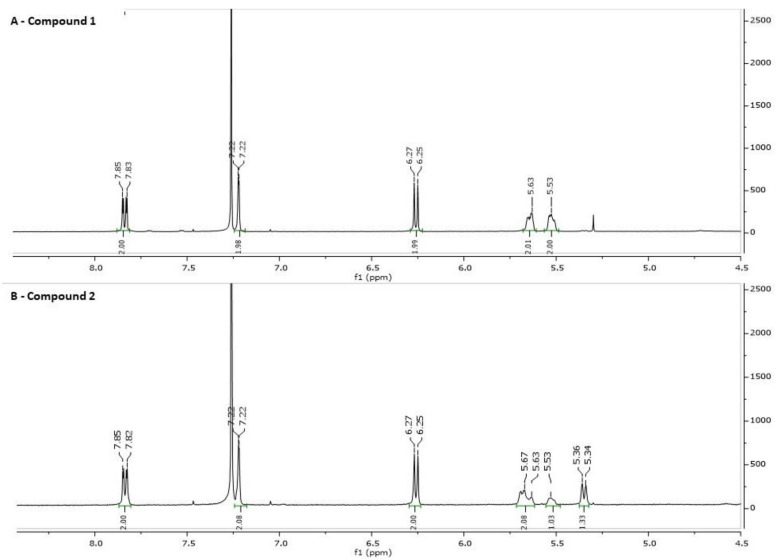
Proton NMR of bufalin 2,3-ene (Compound 1, **A**) and bufalin 3,4-ene (Compound 2, **B**). The compounds at retention time 24–25 min and 25–26 min in the HPLC were collected dried and dissolved in CDCl3 (1.0 mg/mL). Measurements were made with a Bruker AVANCE III HD 500 MHz spectrometer in CDCl3.

**Figure 4:**
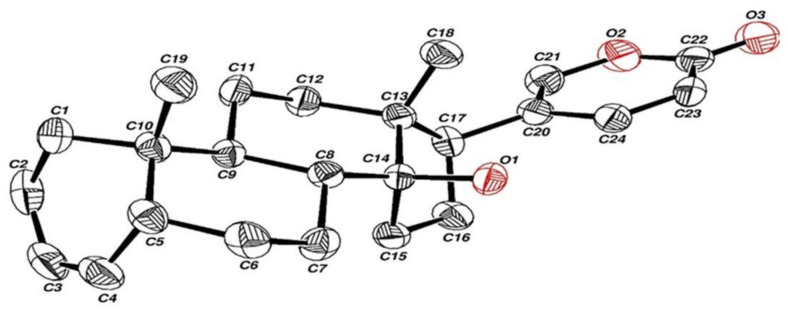
X-ray crystallography of compound 2. After collecting compound 2 (retention time 25–26 min in HPLC), the sample was evaporated and the product was crystalized from a 70% acetonitrile/water solution. X-ray crystallography was obtained with an ENRAF-NONIUS CAD-4 computer-controlled diffractometer.

The proton NMR spectra of the two compounds (Figure 3A,B) supports this conclusion. The NMR spectra of compound 1 (Figure 3A) showed, in addition to the vinyl hydrogens of the diolone ring, two symmetrical vinyl hydrogens at 5.3 and 5.5 ppm, corresponding to a bufalin dehydroxy-2,3-ene isomer. The proton NMR of compound 2 (Figure 3B) shows also the vinyl hydrogens with a clean doublet at 5.3 ppm corresponding to bufalin 3,4-ene. Compound 2 was crystalized from acetonitrile/water 70% and subjected to X-ray crystallography measurements. CCDC 2162504 contains the supplementary crystallographic data for compound. These data can be obtained free of charge from The Cambridge Crystallographic Data Centre via www.ccdc.cam.ac.uk/structures,accessed on 27 March 2022. The interpretation of these measurements is consistent with the structure, bufalin dehydroxy-3,4-ene (Figure 4), with a double bond between the C3 and C4 atoms. This water elimination suggests an unusual catalytic effect induced by Ishikawa’s reagent used to induce the elimination of the 3-OH group to create selectively the double bonds in ring A, the bufalin dehydroxy isomers. All attempts to crystalize compound 1 proved unsuccessful; however, as mentioned above, the two symmetrical vinyl protons support the structure of bufalin 2,3-ene isomer for this compound. 

**Figure 5:**
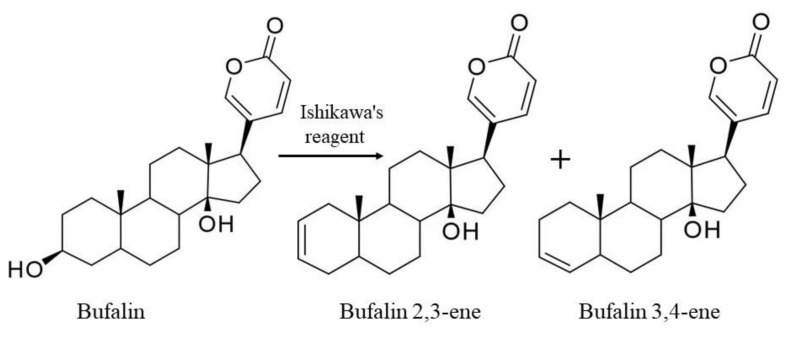
Chemical structures of bufalin, bufalin 2,3-ene, and bufalin 3,4-ene.

The cumulative findings led us to conclude that the structures of the products are bufalin 2,3-ene (compound 1) and bufalin 3,4-ene (compound 2), as depicted in Figure 5.

### 2.2. Biological Evaluation

**Figure 6:**
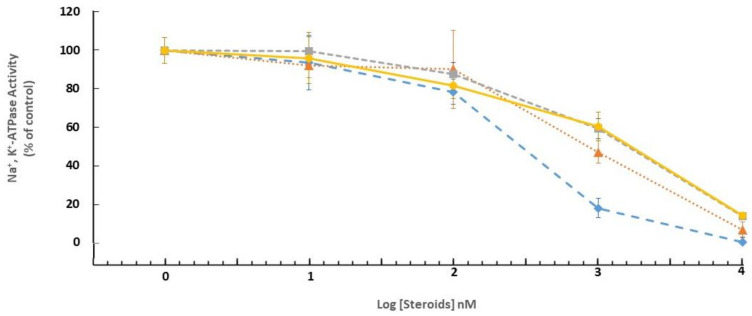
Effect of CS on brain microsomal Na^+^, K^+^-ATPase activity. A pig brain microsomal fraction was exposed to different concentrations of the steroids; reactions (37 °C, 30 min) were terminated by the addition of TCA. The inorganic phosphate content of the samples was determined as described in Materials and Methods. The results are expressed as % Na^+^, K^+^-ATPase activity (4.72 µmol Pi/µg protein/h.), representing 81% of the total ATPase activity (5.78 µmol Pi/µg protein/hr.) in the absence of steroids. The effects of bufalin (blue, ♦), Bufalin 2,3-ene (orange, ▲), bufalin 3,4-ene (grey, ■), and ouabain (yellow, ●) are shown. Values are expressed as the mean ± SE (error bars) of 3 experiments.

The biological characterization of the novel compounds was addressed in systems known to be affected by CS, namely Na^+^, K^+^-ATPase inhibition, cell viability, and heart muscle cell contractility. ATPases activity of pig microsomal fraction was measured by determining phosphate release from ATP in the presence and absence of ouabain (1 mM). In this preparation, about 80% of total ATPase activity is ouabain-sensitive, Na^+^, K^+^-ATPase (Supplementary Table S1). The inhibition of Na^+^, K^+^-ATPase in a pig microsomal fraction by different cardiac steroids is shown in Figure 6. The inhibition curves of all the steroids spanned more than two log units, indicating the established presence of three isoforms of the enzyme in brain tissue. Differences in the potency to inhibit Na^+^, K^+^-ATPase between the steroids were negligible. Bufalin was the most potent (IC_50_ = 26 × 10^−8^ M), bufalin 2,3-ene and bufalin 3,4-ene exhibited a lower potency (IC_50_ = 70 × 10^−8^ and 140 × 10^−8^ M, respectively), and ouabain–the lowest (IC_50_ = 145 × 10^−8^ M). 

**Figure 7:**
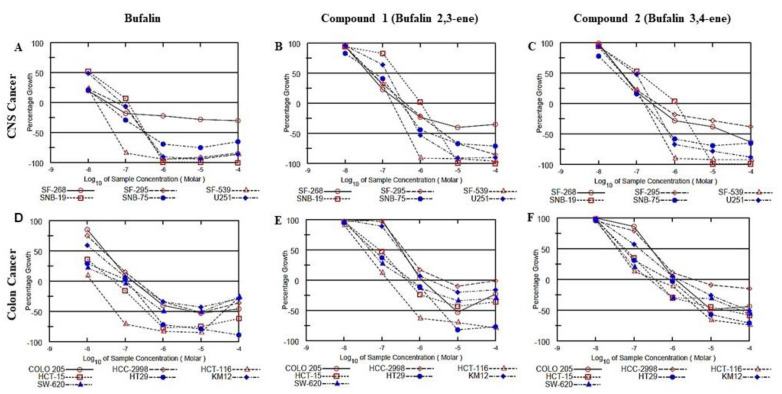
Effect of bufalin, bufalin 2,3-ene, and bufalin 3,4-ene on human cancer cell growth. The anti-proliferative activity of the steroids was tested against the NCI-60 cell line panel at the National Cancer Institute (NCI), Bethesda, Maryland, USA according to their standard protocol (https://dtp.cancer.gov/discovery_development/nci-60/methodology.htm, 27 March 2022). The effects of bufalin and the novel bufalin derivatives on 6 glioblastoma cell lines (**A**–**C**) and 7 colorectal cancer cell lines (**D**–**F**) are shown.

**Figure 8:**
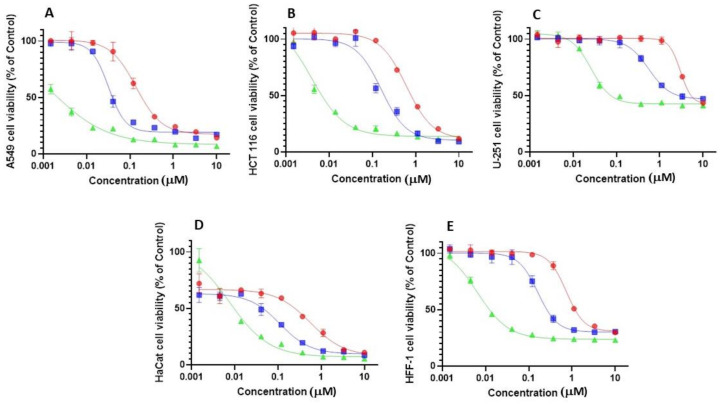
Effect of bufalin, bufalin 2,3-ene, and bufalin 3,4-ene on human cancer and non-cancer cell growth. A549, alveolar basal epithelia cell carcinoma (**A**), HCT 116, colorectal carcinoma (**B**), U-251, glioblastoma (**C**), HaCaT, immortalized keratinocytes (**D**), and HFF-1, fibroblasts (**E**) cell lines were grown as described in Materials and Methods. The cells were incubated in growth media containing different concentrations of bufalin (green, ▲), bufalin 2,3-ene (red, ●), or bufalin 3,4-ene (blue, ■) for 48 h. At the end of incubation period, cell viability was determined as described in Materials and Methods. Percentage of cell viability was calculated using the formula: (fluorescence of treated cells − mean background fluorescence) × 100/(fluorescence of cells with vehicle control (0.33% DMSA) − mean background fluorescence.

The anti-proliferative activity of the novel steroids versus that of bufalin was tested against the NCI-60 cell line panel at the National Cancer Institute (NCI), Bethesda, MD, USA). As an example, Figure 7 depicts the anti-proliferative effects of the steroids on two groups: CNS cancer (6 glioblastoma cell lines) and colon cancer cell lines (7 colorectal carcinoma cells with different morphological characteristics). The complete analyses on the 60 cell lines are available in Supplementary Figure S1. In all the cells examined the anti-proliferative activity of bufalin 2,3-ene and bufalin 3,4-ene was weaker than that of bufalin. Whereas, in most cases 50% inhibition of cell growth by bufalin was obtained in the range of 10^−7^ M, about tenfold higher concentrations were required for bufalin 2,3-ene and bufalin 3,4-ene (Figure 8 and Supplementary Figure S1), indicating the lower toxicity of the new compounds.

To explore the relative toxicity of the bufalin derivatives to tumor and non-tumor cells, a separate experiment was performed in which the effects of the steroids on viability was tested on three human cancer cell lines (A549, alveolar basal epithelia cell carcinoma, HCT 116, colorectal carcinoma, and U-251, glioblastoma) and two human, non cancer cell lines (HaCaT, immortalized keratinocytes and HFF-1, fibroblasts). In agreement with the results described above on the NCI-60 cell line panel, bufalin was a more potent inhibitor than bufalin 2,3-ene and bufalin 3,4-ene of cell viability in all cells tested (Figure 8). Furthermore, none of the steroid tested showed a significant increased toxicity on the cancer cells compared to that on non-cancer cells. 

**Figure 9:**
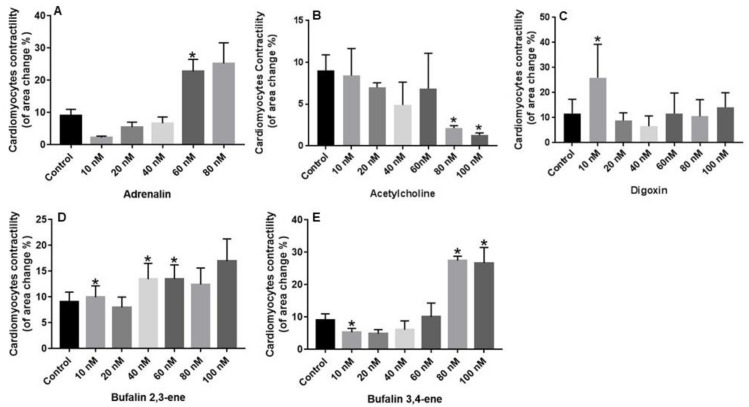
Effect of bufalin-2,3-ene and bufain-3,4-ene on quail cardiac cell contractility. Quail cardiomyocyte preparation and contractility measurements were performed as described in Materials and Methods. Contractility at 37 °C was measured 30 min after drug addition. Cells were photographed for 15 s, and the change in cell contraction was deduced from the mean difference in area change between relaxation and the contraction peaks. Three cell clusters in 3 wells were photographed and measured for each experimental condition. Quantification of motility is presented as cell area change. The effect of adrenalin (**A**), acetylcholine (**B**), digoxin (**C**), bufalin 2,3-ene (**D**), and bufalin 3,4-ene (**E**) at different concentrations is shown. Bar graphs represent the mean ± SE. of 3 experiments. Values significantly different from that of the control =, * *p* < 0.05.

Quails are frequently used for the study of heart and coronary artery development [21] and primary cultured cardiomyocytes from this species serve to study the regulation of vertebrate early heart morphogenesis [22]. In the present study the cardiotonic effect of the novel bufalin 2,3-ene and bufalin 3,4-ene was determined by measuring their influence on the contractility of quail primary cardiomyocytes from E4 embryos in culture. The quail and their embryos were sacrificed and used in other experiments and their convenient availability prompted us to use them in the present study. First, to validate the cardiomyocyte contractility measurements, we tested the effects of adrenergic and cholinergic agonists on the cell contractility of quail cardiomyocytes. As expected, treatment of quail cardiomyocytes with noradrenalin increased dose-dependently the force of contraction, conversely, exposure of the cells to acetylcholine, dose-dependently reduced the force of contraction (Figure 9A,B). Addition of the CS digoxin to the cell media also increased the force of cardiomyocyte contraction at 10 nM, demonstrating their suitability for basic pharmacological screening of CS effects. Primary cardiomyocytes from E2 and E3 embryos did not display increased contractility following digoxin treatment (data not shown). The effects of bufalin 2,3-ene and bufalin 3,4-ene on quail cardiomyocyte contractility is depicted in Figure 9D,E. Although bufalin 2,3-ene showed positive inotropy at lower concentrations (40 nM), the effect of bufalin 3,4-ene was more robust, demonstrating a more than threefold increase in contractility at 80 nM (Figure 9E). The effect of the two compounds on contractility was greater than that of digoxin: whereas at 100 nM, digoxin increased the force of contraction by 22%, increases of 105% and 194% were obtained with bufalin 2,3-ene and bufalin 3,4-ene, respectively (Figure 9).

## 3. Discussion

In the present study we describe the organic synthesis of two novel CS: bufalin 2,3-ene and bufalin 3,4-ene and evaluated their biological effects by testing their ability to inhibit Na^+^, K^+^-ATPase activity, to induce cytotoxicity on cancer cells and to increase cardiomyocytes contractility. 

CS bufalin is the most extensively investigated, active ingredient of a traditional Chinese medicine Chan-Su obtained from the parotid venom glands and skin of a toad. The compound has been shown to exert numerous anti-tumor effects in lung, colorectal, gastric, and liver cancer [23] and exhibits anti-inflammatory and anti-viral activities [8,9,10]. Furthermore, bufalin proved to be more effective than other CS in inhibiting ion transport by Na^+^, K^+^-ATPase and [24,25] and a more potent vasoactive agent than ouabain [26,27]. Importantly, as described above, due to the toxic manifestations of bufalin, like other CS, the therapeutic window of this steroid is extremely small. Therefore, the impressive pharmacological repertoire of bufalin and the objective of finding a less toxic CS, prompted us to synthesize derivatives of this steroid and evaluate their biological effects. 

The organic synthesis of the two steroids bufalin 2,3-ene and bufalin 3,4-ene was achieved by using Ishikawa’s reagent, conventionally used to convert alcohols into fluorides. The reagent is used mainly to convert primary alcohols into alkyl fluorides under very mild conditions, resulting in a very high yield. However, secondary alcohols, such as sterols, in addition produce alkenes and ethers as side products [20]. Apparently, as bufalin is a secondary alcohol, such water elimination occurs, resulting in selective removal of the 3-OH group to create the double bonds in ring A, i.e., the bufalin 2,3-ene and bufalin 3,4-ene isomers.

We demonstrated that the two steroids manifest several of the biological effects attributed to CS, including inhibition of ATP hydrolysis by Na^+^, K^+^-ATPase, inhibition of cancer cell growth and increased contraction of heart muscle cells. However, the two compounds differ in some features, which may be of relevance for their potential development as drugs. 

Since all the CS inhibited Na^+^, K^+^-ATPase activity (Figure 6), the difference in the positive cardiotonic effects by the steroids cannot be attributed to differences in pump inhibition, but to other molecular mechanisms. A substantial body of studies has demonstrated that CS also act by directly and indirectly affecting the activity of the nuclear receptor superfamily of transcription factors [28,29]. In their pioneering study, Wang et al. showed that CS inhibit the level of the steroid receptor coactivator -3 protein (SRC-3) transcription factor by directly binding to this protein [29]. There were significant differences between the CS in inducing SRC-3 activity inhibition and bufalin was found to be the most potent of all the compounds tested. It is possible that the differences in CS-induced increase in contractility are attributable to the discrepancies in their effect on the transcription factors. 

Direct experimental evidence for the anti-cancer effects of CS, and bufalin in particular, was reported forty years ago [30,31]. Structure-activity determination, with ATP hydrolysis by Na^+^, K^+^-ATPase as activity determinant, pointed to the significance of the 3 β-hydroxyl group of the steroid [32]. In a more detailed study, CS with attached ethylene glycol moieties of varying length at the 3 β-hydroxyl position were evaluated for their potency to inhibit Na^+^, K^+^-ATPase and for their cytotoxic effect on cancerous MCF-7 cells [18]. A clear trend was observed in both parameters, namely, a decrease in bioactivity as size increases. Notably, compounds lacking the OH residue at the 3 β position were not available and, therefore, not tested. Our current results showed that omission of the OH group not only did not increase the toxicity of the steroids, but reduced it by about tenfold (Figure 8, Supplement Figure S1). This implies that, as in Na^+^, K^+^-ATPase inhibition, the 3-OH group at the 3-position is essential for activity and that the novel compounds are not good candidates for development as anti-cancer drugs. Furthermore, the lack of a relative increased toxicity of the steroids on cancer cells compared to non-cancer cells also supports this conclusion. 

The inotropic effect of the compounds was tested on quail primary cardiomyocytes (Figure 9). The mature chick heart includes four chambers with in- and out-flow tracts and, despite some differences, for example, during septation and aortic arch remodeling, it resembles the human heart more closely than that of other non-mammalian model organisms. Owing to those features, and the available toolkit described below, avian embryos will almost certainly continue to contribute significant insights into the development and function of the heart. The short (16 days) developmental period of this species and its readily accessible embryo made it a convenient animal model in numerous fields of research, including developmental biology, endocrinology, aging, immunology, behavior studies, and a variety of human genetic disorders, but it was scarcely used for pharmacological screening [33]. We, therefore, first characterized the effects of cardioactive drugs, adrenalin and acetylcholine on the contractility of quail cardiomyocytes. As expected, adrenalin increased and acetylcholine decreased the force of contraction of the cardiomyocytes (Figure 9A,B). Digoxin at 10 nM increased the force of contraction, but had no effect on contractility at higher concentrations, presumably due to the toxic effects of this steroid at higher concentrations [34,35]. We found that at 80–100 nM, bufalin 2,3-ene and bufalin 3,4-ene increased the force of contraction of quail primary cardiomyocytes in culture (Figure 9D,E). No effect was observed lower concentrations. Clearly, such an effect merits further testing in mammalian experimental systems and, eventually, in the human heart. The potential for a compound to serve as an inotropic drug depends on its therapeutic index, the range of doses at which a compound increases the force of contraction without unacceptable adverse events. Importantly, despite the lower potency in increasing contractility, the reduced cytotoxic effect of the two novel steroids (Figure 9) advocate for their development as drugs for the treatment of heart failure. 

## 4. Materials and Methods

### 4.1. Materials 

Solvents were purchased from Romical (Jerusalem, Israel), bufalin and Ishikawa’s reagent were purchased from Chengdu Biopurify Phytochemicals Ltd. Wenjiang, Chengdu, China and Sigma Aldrich Co. (St. Louis, MO, USA), respectively. TLC Silica Gel 60 F254 Aluminum Sheets were purchased from Merck, (Darmstadt, Germany) and C18 columns from Phenomenex, Torrance, CA, USA. A549, alveolar basal epithelia cell carcinoma, HCT 116, colorectal carcinoma, and HFF-1, human fibroblasts were obtained from ATCC, (Manassas, VA, USA). HaCaT, immortalized keratinocytes were obtained from AddexoBio (San Diego, CA, USA) and U251 glioblastoma cells were from ECACC General Cell Collection (Salisbury, United Kingdom). Serum, DMEM cell culture medium, antibiotics, and a chemiluminescence kit were acquired from Biological Industries (Beit Ha’emek, Israel). ATP and protease inhibitor cocktail were purchased from Sigma-Aldrich, (St. Louis, MO, USA). Pierce primary cardiomyocyte isolation kit was obtained from Thermo Scientific, Rockford, IL, USA.

### 4.2. High Performance Liquid Chromatography 

HPLC was performed with a Hewlett-Packard (HP) 1050 Series chromatograph with a HP 1010 detection system and an Agilent computer system. A 50 µL volume of each sample was passed through a Luna C-18, 5 μm column (250 × 4.6 mm) (Phenomenex, Torrance, CA, USA), provided with a pre-column. Elution was performed with a 35 min, 68% CH_3_CN/water isocratic system with a flow rate of 1.0 mL/min. 

### 4.3. NMR and Mass Spectroscopy

The 1H NMR (500 MHz) measurements were performed with a Bruker AVANCE III HD 500 Mhz spectrometer in CDCl3. Electron spray mass spectra were obtained with a Quadrupole LCMS mass spectrometer system (Thermo Scientific, Mundelein, IL, USA). 

### 4.4. Crystallographic Structure Analysis 

Crystallography was performed on an ENRAF-NONIUS CAD-4 computer-controlled diffractometer, and all crystallographic computing was made with a VAX9000 computer at the Hebrew University of Jerusalem. 

### 4.5. Chemical Experimental Procedures

A *Bufalin-2,3-ene (compound 1)*. A suspension of bufalin (15 mg, 0.039 mmol), in dry diethyl ether (1.0 mL) was prepared in a 5 mL glass vial with a magnetic stirrer. The vial was cooled at 8 °C, wrapped in aluminum foil and Ishikawa’s reagent (70 mg, 0.310 mmol, 60 μL) was added slowly. The mixture was stirred for 2 h at 8 °C and overnight at room temperature. To separate the product from excess reagent, the solution was loaded on a silica gel thin-layer chromatography (TLC) plate and eluted with 70% diethyl ether and hexane for 4 min. Following visualization with a UV lamp at 260 nm, the silica gel plate at 2 cm was scraped into a small vial and the organic material was dissolved in methanol (0.3 mL). A clear suspension from this solution (50 μL) was injected into the high-performance liquid chromatography (HPLC) system, producing the pattern shown in Figure 1. The peak with an absorbance at λ max 300 nm at 24–25 min retention time was collected and dried to afford compound 1 (0.25 mg, 1.7% yield. MS: *m*/*z*/(MH+) 369. 1H NMR: δ 7.82 (d, 1H, H-C [22]), 7.22 (s, 1H, H-C [21]), 6.24 (d, 1H, H-C [23]), 5.63 (m, 1H, H-C [2]) 5.53 (m, 1H, H-C [3) 0.95 (s, 3H, CH3 [19]) 0.70 (s, 3H, CH3 [18]). 13C NMR: δ 16.69 (C18), 22.47 (C19), 85.93 (C14), 51.42 (C17)122.88 (C20), 148.62 (C21), 146.94 (C22), 115.46(C23), and 162.48 (C24).

*Bufalin-3,4-ene (compound 2).* Following the above procedure, the peak that had a UV spectrum at λ max 300nm at 25–26 min retention time was collected and dried, afforded compound 2 (0.55 mg, 3.5% yield). MS: *m*/*z*/(MH+) 369. 1H NMR: δ 7.83 (d, 1H, H-C [23]), 7.22 (s, 1H, H-C [21]), 6.25 (d, 1H, H-C [22]), 5.68 (m, 1H, H-C [3]) 5.34 (d, 1H, H-C [4]) 0.94 (s, 3H, CH3 [19]) 0.71 (s, 3H, CH3 [18]). 13C NMR: δ 16.71 (C18), 21.83 (C19), 83.93 (C14), 51.47 (C17), 127.46 (C20), 148.62 (C21), 146.94 (C22), 115.46 (C23), and 162.54 (C24).

### 4.6. ATPase Activity

A pig brain microsomal fraction was prepared as previously described [36]. Na^+^, K^+^-ATPase activity in the microsomal fraction was determined by the amount of inorganic phosphate released during incubation at 37 °C. In brief: 480 µL of the microsomal preparation (60 µg protein) was added to 3520 µL reaction buffer (50 mM Tris-Base, 120 mM NaCl, 10 mM KCI, 4 mM MgCl_2_, pH 7.4) in the presence of varying concentrations of an inhibitor (bufalin or bufalin derivatives). Following 20 min incubation, 10 µL of ATP (2.5 mM final concentration) was added and the incubation was allowed to proceed for an additional 30 min. The reaction was terminated by the addition of 1 mL 16% trichloroacetic acid and placing the tubes on ice for 10 min. Following centrifugation (500× *g*, 10 min, 4 °C), 50 µL of the supernatant was removed for determination of inorganic phosphate according to a colorimetric method, as described previously [37].

### 4.7. Cytotoxicity against Cancer Cells

The anti-proliferative activity of the target compounds was tested against the NCI-60 cell line panel. The screening was performed at the National Cancer Institute (NCI), Bethesda, MD, USA (www.dtp.nci.nih.gov, 20 March 2020), according to their standard protocol (https://dtp.cancer.gov/discovery_development/nci-60/methodology.htm, 20 March 2020). 

All cell lines were grown at 37 °C in 5% CO_2_. A549 cell lines were grown in F-12 (Ham’s) media with L-Glutamine supplemented with 10% FBS, 1% sodium pyruvate, and 1% Penicillin–Streptomycin. HCT 116 cell line were grown in McCoy’s 5A Medium with L-Glutamine, supplemented with 10% FBS, 1% sodium pyruvate, and 1% Penicillin–Streptomycin. U-251 cell line were grown in MEM-NEAA, Earle’s Salts Base, with Non-Essential Amino Acids, supplemented with 10% FBS, 1% sodium pyruvate, 1% L-Alanyl-L-Glutamine, 0.01 mg/mL human recombinant insulin, and 1% Penicillin–Streptomycin. HaCaT and HFF-1 cell lines were grown in DMEM with L-Glutamine, supplemented with 10% FBS, 1% sodium pyruvate, and 1% Penicillin–Streptomycin.

One day before the experiments, cells from all cell lines were seeded into 96-well plates at a density of 1 × 10^4^ cells/well in 100 µL of appropriate growth media. Plates were incubated overnight in 37 °C to allow attachment. Growth media (100 µL) containing the tested steroids were added to obtain the desired concentration and the cells were incubated for 48 h in 37 °C. Cells incubated in growth media containing 0.33% DMSO (vehicle) served as control. At the end of incubation period, cell viability evaluation was performed using VisionBlue™ Quick Cell Viability Fluorometric Assay Kit, according to manufacturer’s instructions. Fluorescent signal by was detected by TECAN spark 10 M microplate reader, Excitation/Emission 535 ± 25/590 ± 20 nm. The fluorescence data are expressed as percentage of cell viability (%) compared to vehicle control.

### 4.8. Quail Cardiac Muscle Cell Contractility

Quantification of cardiomyocytes contractility was performed as previously described [35]. Since avian embryos (quail included) are not considered “animals”, their use is exempt at the Hebrew University, like in all other academic institutions, of the need for ethical approval. Quail (*Coturnix japonica*) cardiomyocytes were prepared from E4 embryos with a Pierce Primary Cardiomyocyte Isolation Kit (Thermo Scientific™). Contractility at 37 °C was measured 30 min after drug addition. Cells were photographed for 15 sec, with an Olympus CKX41 (Japan) upright microscope (×20 magnification), and integrated incandescent illumination. A FastCam imi-tech (Gyeonggi-do, South Korea) high speed digital camera with a 640 × 480 pixel, gray scale image sensor was mounted on the microscope with ImCam software (IMI Tecnology, Co., Ltd., Gangnam-gu, Seoul, Korea). Changes in cell contraction were deduced from the mean difference in area change between relaxation and the contraction peaks [35]. Three cell clusters in three wells were photographed and measured under each experimental condition.

### 4.9. Statistical Analysis

All the data are expressed as the mean ± standard error (SEM). Significance was determined according to the independent Student’s *t*-test; *p* < 0.05 was considered significant.

## Data Availability

Not applicable.

## Supplementary Materials

The following supporting information can be downloaded at https://www.mdpi.com/article/10.3390/ijms23074007/s1.
