# Supplementary material for: Synthesis and Biological Evaluation of Novel Bufalin Derivatives
Source: Int J Mol Sci. 2022 Apr 4;23(7):4007. doi: 10.3390/ijms23074007 (PMC8999407; doi:10.3390/ijms23074007)
Supplement: Supplementary file 1 [file ijms-23-04007-s001.zip › Supplement 1.pdf]

Supplement 1.

Effect of bufalin, bufalin 2,3-ene and bufalin 3,4-ene on ATPases activity

	Total ATPase	Na^+, K^+-ATPase	Mg^{++}-ATPase
	μmole Pi/mg Protein/Hour		
Control	6.60	5.31	1.28
Bufalin (10 μM)	6.55	5.08	1.47
Bufalin 2,3-ene (10 μM)	6.11	4.57	1.54
Bufalin 3,4-ene (10 μM)	5.52	4.31	1.21

ATPases activity of pig microsomal fraction was measured by determining phosphate release from ATP in the presence and absence of ouabain (1 mM), as described in Materials and Methods.
