# Supplementary material for: Synthesis and Biological Evaluation of Novel Bufalin Derivatives
Source: Int J Mol Sci. 2022 Apr 4;23(7):4007. doi: 10.3390/ijms23074007 (PMC8999407; doi:10.3390/ijms23074007)

Supplement 2.

Effect of bufalin, Compound 1 and Compound 2 on human cancer cells growth. The anti-proliferative activity of the steroid was tested against the NCI-60 cell line panel at the National Cancer Institute (NCI), Bethesda, Maryland, USA applying their standard protocol (https://dtp.cancer.gov/discovery_development/nci-60/methodology.htm).

Figure S1. Effect of Bufalin on human cancer cell growth.

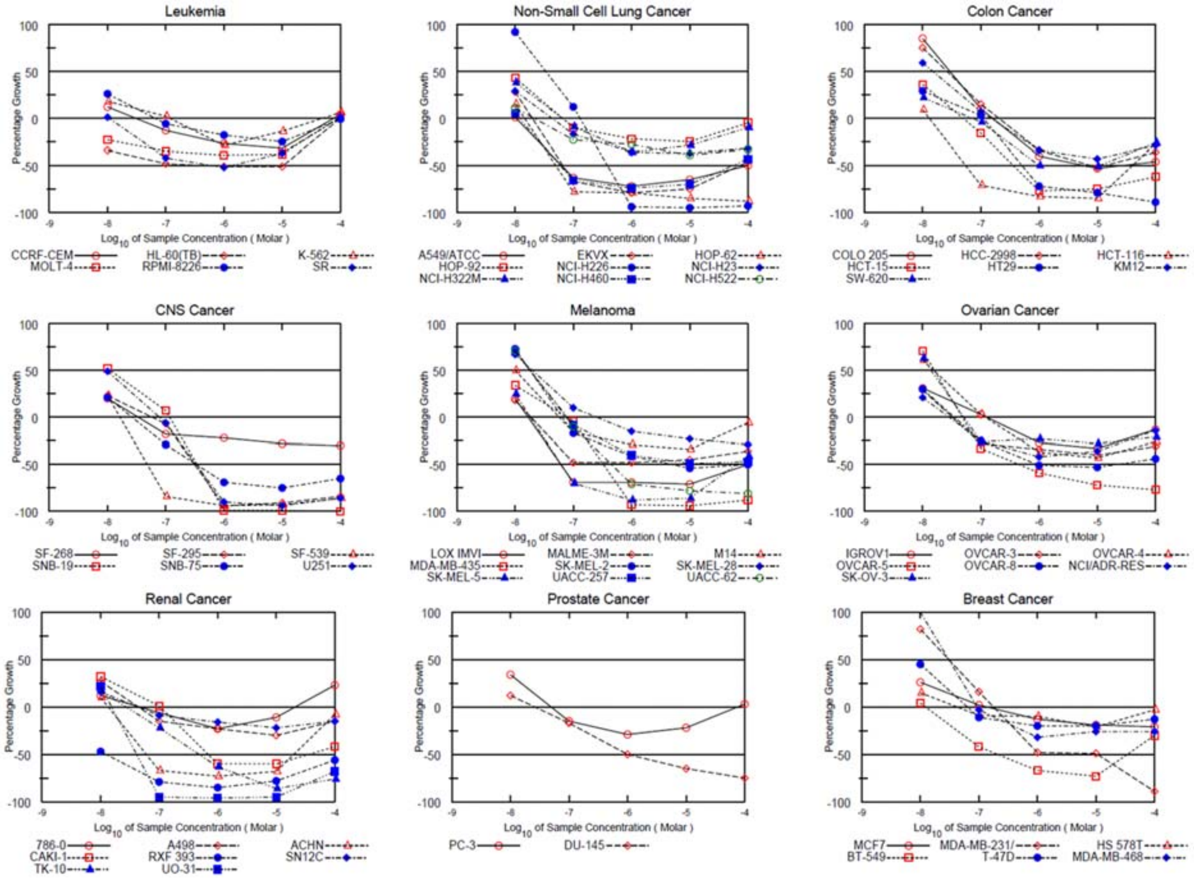

Figure S2. Effect of Compound 1 (Bufalin 2,3-ene) on human cancer cell growth

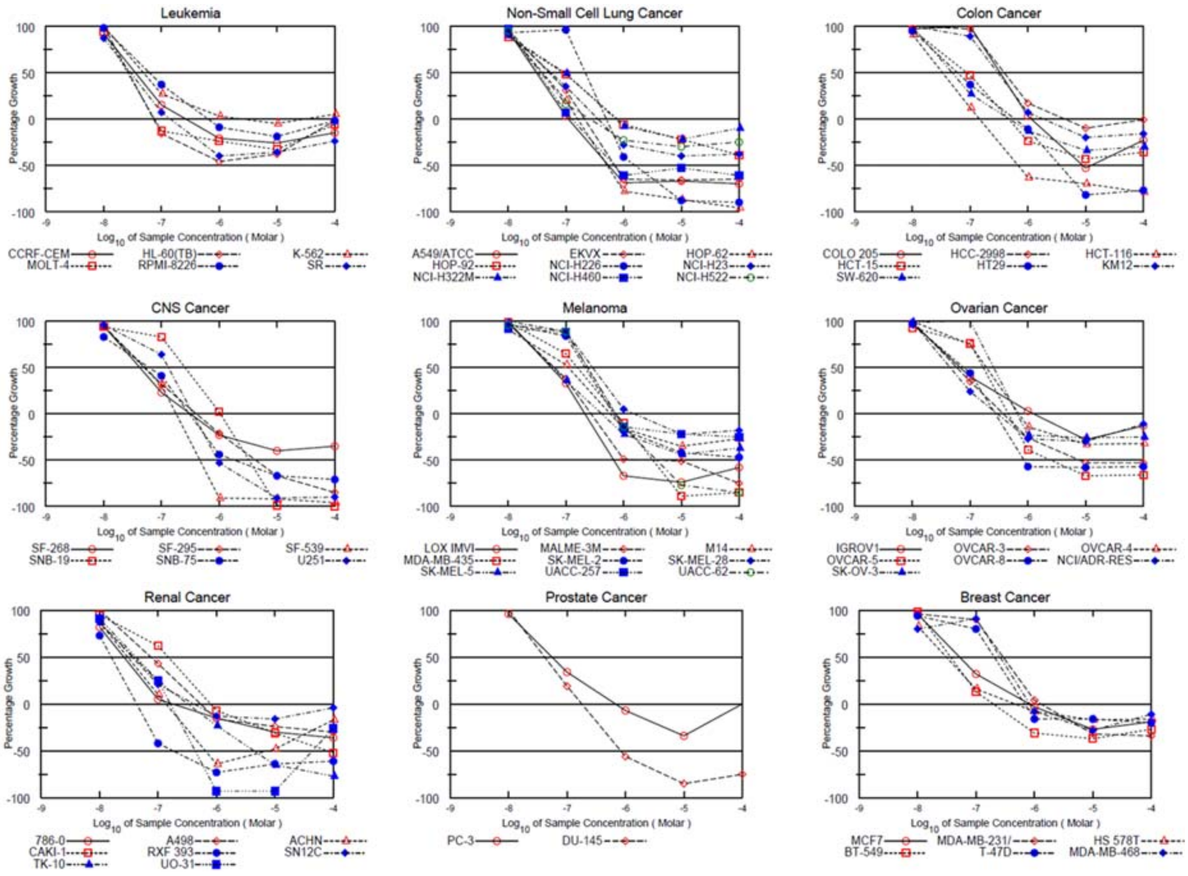

Figure S3. Effect of Compound 2 (Bufalin 3,4-ene) on human cancer cell growth

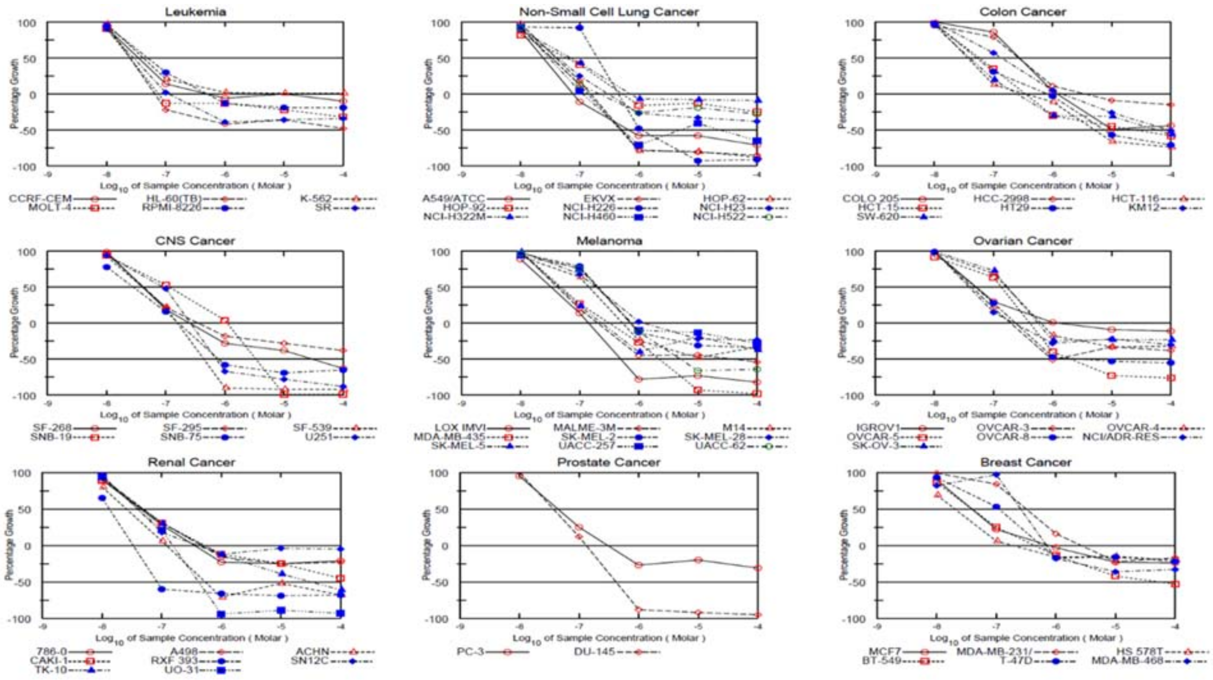

Supplement: Supplementary file 1 [file ijms-23-04007-s001.zip › Supplement 2.pdf]
